# Comparison of coproprevalence and seroprevalence to guide decision-making in national soil-transmitted helminthiasis control programs: Ethiopia as a case study

**DOI:** 10.1371/journal.pntd.0010824

**Published:** 2022-10-05

**Authors:** Sara Roose, Gemechu Tadesse Leta, Johnny Vlaminck, Birhanu Getachew, Kalkidan Mekete, Iris Peelaers, Peter Geldhof, Bruno Levecke

**Affiliations:** 1 Department of Translational Physiology, Infectiology and Public Health, Ghent University, Merelbeke, Belgium; 2 Bacterial, Parasitic and Zoonotic Diseases Research Directorate, Ethiopian Public Health Institute, Addis Ababa, Ethiopia; Natural History Museum, UNITED KINGDOM

## Abstract

**Background:**

WHO recommends periodical assessment of the prevalence of any soil-transmitted helminth (STH) infections to adapt the frequency of mass drug administration targeting STHs. Today, detection of eggs in stool smears (Kato-Katz thick smear) remains the diagnostic standard. However, stool examination (coprology) has important operational drawbacks and impedes integrated surveys of multiple neglected tropical diseases. Therefore, the aim of the present study was to assess the potential of applying serology instead of coprology in STH control program decision-making.

**Methodology:**

An antibody-ELISA based on extract of *Ascaris* lung stage larvae (AsLungL3-ELISA) was applied in ongoing monitoring activities of the Ethiopian national control program against schistosomiasis and soil-transmitted helminthiasis. Blood and stool samples were collected from over 6,700 students (median age: 11) from 63 schools in 33 woredas (districts) across the country. Stool samples of two consecutive days were analyzed applying duplicate Kato-Katz thick smear.

**Principal findings:**

On woreda level, qualitative (seroprevalence) and quantitative (mean optical density ratio) serology results were highly correlated, and hence seroprevalence was chosen as parameter. For 85% of the woredas, prevalence based on serology was higher than those based on coprology. The results suggested cross-reactivity of the AsLungL3-ELISA with *Trichuris*. When extrapolating the WHO coproprevalence thresholds, there was a moderate agreement (weighted κ = 0.43) in program decision-making. Using the same threshold values would predominantly lead to a higher frequency of drug administration.

**Significance:**

This is the first time that serology for soil-transmitted helminthiasis is applied on such large scale, thereby embedded in a control program context. The results underscore that serology holds promise as a tool to monitor STH control programs. Further research should focus on the optimization of the diagnostic assay and the refinement of serology-specific program decision-making thresholds.

## Author summary

Worldwide, children are treated with deworming drugs to control the morbidity caused by intestinal worms. These drugs are provided at school through large-scale deworming programs. The frequency of deworming in a certain administrative area (e.g. a district) is based on the prevalence (proportion of children testing positive for worm infections) in that particular area. The current diagnostic standard is the microscopic detection of worm eggs in a stool smear. However, this technique has a lot of drawbacks (e.g. time-consuming, need for skilled microscopists), and makes it impossible to integrate in control programs targeting different neglected tropical diseases. A potential alternative method is serology, the detection of antibodies against worms in the blood of children. We analysed blood and stool samples for more than 6,700 children from 33 districts in Ethiopia. On district level, the seroprevalence (proportion of children having antibodies) was generally higher than the coproprevalence (proportion of children shedding worm eggs). Decisions about the frequency of deworming based on both tests showed a moderate agreement. The study is a proof-of-concept that serology holds promise as an alternative tool to monitor deworming programs. Further research should focus on the optimization of the serological assay and the refinement of serology-specific thresholds for determining deworming frequencies.

## Introduction

Recently, the World Health Organization (WHO) published its 2021–2030 roadmap for neglected tropical diseases (NTDs) [1]. With this document the WHO aims to ensure continued support for the control of NTDs for the next decade, with the ultimate goal to steer stakeholders towards four global targets: (1) reduce the number of people requiring interventions against NTDs by 90%, (2) decrease the global disease burden with 75%, (3) eliminate at least one NTD in 100 countries and (4) eradicate two NTDs by the end of 2030 [1]. To assess the progress towards these ambitious 2030 targets, periodic monitoring and evaluation (M&E) surveys remain crucial. Although detailed M&E survey guidelines are developed for 15 of the 20 NTDs, providing recommendations on study design, diagnostic tests and program decision rules [2], there is an urgent need for diagnostic platforms with improved performance that enable the screening of a large number of individuals for multiple NTDs using a single sample [3,4].

To date, most NTD programs rely on the microscopic examination of a wide range of sample types such as stool (e.g. STHs and intestinal schistosomiasis), urine (e.g. urinary schistosomiasis), blood (e.g. lymphatic filariasis), skin scrapings or needle aspirates (e.g. cutaneous leishmaniasis) and skin biopsies (e.g. onchocerciasis). Therefore, the currently used tests are prone to human-error, have a relatively low throughput rate and also impede an integrated M&E of multiple NTDs programs [3,4]. As a response to this gap in the diagnostic armamentarium, the WHO has recently established the Diagnostic Technical Advisory Group (WHO-DTAG) with the purpose to identify the diagnostic needs for NTDs and to define the minimal and ideal characteristics for new diagnostics that intent to guide NTD programs—the so-called target product profiles (TPPs) [5–8]. Currently, TPPs have been developed for four NTDs that are emendable through large-scale deworming programs, including lymphatic filariasis [9], onchocerciasis [10], schistosomiasis [11] and soil-transmitted helminthiasis [12]. For these four diseases, the only mutual recommended sample specimen is peripheral whole-blood obtained by finger prick [9–12]. Blood-based diagnostics are currently commercially available for lymphatic filariasis and onchocerciasis [9,10]. Though for STHs and schistosomiasis, implementation of new blood-based diagnostic tools in the field within the time frame of the 2030 roadmap is unlikely considering the barriers that exist to biomarker discovery and test development [13]. These challenges however stress the need for continued research to enable implementation of non-stool-based diagnostic platforms beyond the 2030 WHO roadmap, facilitating stop intervention decisions and post-program surveillance.

Soil-transmitted helminthiasis accounts for 12% of the total disease burden attributed to NTDs and is caused by a group of intestinal worms (soil-transmitted helminths, STHs) including *Ascaris lumbricoides*, *Trichuris trichiura*, *Ancylostoma duodenale*, *Necator americanus* and *Strongyloides stercoralis* [14]. The WHO published six 2030 targets and corresponding milestones for soil-transmitted helminthiasis, of which the first two are based on large-scale epidemiological surveys [15]. Target #1 is to achieve and maintain elimination of STH-attributable morbidity in (pre-)school aged children. Target #2 is to half the global need for anthelmintic tablets (albendazole (400 mg) or mebendazole (500 mg)) in large-scale deworming programs by 2030 [15]. Program decision-making about the frequency of administration of anthelmintic drugs is based on predefined thresholds of any STH prevalence [15]. To assess prevalence, the WHO recommends the microscopic detection of STH specific eggs in stool smears (Kato-Katz thick smear) [16,17]. However, next to the disadvantages of microscopic tests discussed before, an additional issue here is the fact that using eggs as a diagnostic proxy for infection leads to an underestimation of STH prevalence. Only mature worms can be detected after a prepatent period, and egg production varies due to density-dependent factors, male/female worm ratios, geography and immune status of the host [18–23].

Research in veterinary helminthology has shown that detecting anti-parasite antibodies in blood (serology) holds promise as an alternative diagnostic to copromicroscopy, as it allows for the detection of exposure to parasite eggs, larvae and/or adults [24,25]. Two antibody-ELISA (Ab-ELISA) assays (based on (1) *Ascaris suum* haemoglobin (AsHb) and (2) total protein extract of *Ascaris* L3 larvae (AsLungL3)) developed for the use in pigs showed a higher sensitivity than coprology to assess exposure to *Ascaris* infections, and provided more accurate assessments of disease burden (e.g. liver white spots and average daily gain) [24–29]. These assays also showed their potential in humans. Firstly, it was shown that anti-*Ascaris* seroprevalence steadily increased with age in an endemic area in Ethiopia, providing evidence that both children and adults are continuously exposed to *Ascaris*, and that the absence of eggs in the stool of older individuals does not necessarily imply the absence of exposure to larval stages [30]. Secondly, the ELISA-assays were also used to examine the impact of MDA programs; two geographically focalized studies in Ethiopia and Indonesia showed that both *Ascaris* sero- and coproprevalence decreased following MDA [30,31]. More recently, a longitudinal study in 66 Ethiopian children of 4–6 years of age showed exposure to *Ascaris* from a very early age onwards, and a positive correlation between antibody levels and re-infection rate [32]. Considering optimal application of the ELISA assays in humans, it was concluded that the AsLungL3-ELISA performed better than the AsHb-ELISA [30], and that compared to other Ab-isotypes, IgG4 is the Ab-isotype of choice [32]. In the present study, the AsLungL3-ELISA measuring IgG4 levels was applied in ongoing nationwide M&E activities of the Ethiopian national control program against schistosomiasis and soil-transmitted helminthiasis with the main objective to further evaluate the use of STH serology in program decision-making. More specifically, the following 3 research questions were addressed: (1) which parameter should be used to report serology results (seroprevalence or mean optical density ratio), (2) is there cross-reactivity of the AsLungL3-ELISA with other STH species, and (3) can we use serology to guide decision-making in STH control programs.

## Methods

### Ethics statement

Ethical approval of this study was secured by the Ethiopian Public Health Institute Scientific and Ethical Review Office (reference number SERO-128-4-2005; amended to include the blood sampling), and the Institutional Review Board of Imperial College London (reference number ICREC_8_2_2). Official letters were written by the Ethiopian Public Health Institute (EPHI) to the Regional Health and Education Bureaus to obtain regional consent. The school director provided written consent on behalf of the students’ families or guardians as the survey falls under the mandate of the Ministry of Health. Teachers and students were informed about the study in their languages including the purpose, the benefits, the potential risks and the operational procedures. Before sample collection, students provided verbal consent to be included in the survey. Serum samples from the non-endemic adult population in Belgium were acquired through Red Cross Flanders (order numbers: CG2016 0404A, CM2016 0627B and CG2016 1219F).

### Study design

#### Selection of sentinel schools

A cross-sectional survey was embedded in the M&E activities of the Ethiopian national control program against schistosomiasis and soil-transmitted helminthiasis. Since 2016, EPHI, the technical arm of the Ethiopian Federal Ministry of Health, has been annually assessing both prevalence and intensity of STH and *Schistosoma mansoni* infections in 175 sentinel schools across the country. A subset of these schools were selected in two consecutive steps. In a first step, the 175 schools were classified into six levels of endemicity based on the coproprevalence results for *Ascaris* during the national mapping of STHs between 2013 and 2015 (level 1: coproprevalence = 0%; level 2: 0% < coproprevalence < 5%; level 3: 5% ≤ coproprevalence < 10%; level 4: 10% ≤ coproprevalence < 20%; level 5: 20% ≤ copro-prevalence < 30%; level 6: copro-prevalence ≥ 30%) [33]. Second, maps were made using QGIS (version 3.16.16), and 14 geographical clusters of schools with varying levels of *Ascaris* endemicity were purposively selected considering both logistic implications and safety concerns (**S1 Info**) [34]. Since this was an exploratory study, no formal sample size calculations were performed. The final selection included a total of 69 schools across 35 woredas in 7 regional states (Southern Nations and Nationalities Peoples Region (SNNPR), Harari, Gambella, Amhara, Oromia, Benishangul-Gumuz and Tigray). All the selected schools are listed in **S2 Info**, including the initial school endemicity level for *Ascaris* as well as the endemicity of lymphatic filariasis and onchocerciasis on woreda level [35, 36]. In addition to a general training provided by EPHI, the teams involved in the sampling of the selected schools (seven teams for each part of the survey) received an extra training for the purpose of this study on blood sample collection, plasma collection and sample storage. The 38 schools from SNNPR, Harari and Gambella were incorporated in the first part of the national survey (April 2019). The remaining 31 schools in Amhara, Oromia, Benishangul-Gumuz and Tigray were included in the second survey part (November 2019).

#### Sample collection and analysis

A total of 125 students per school were recruited according to the protocol of the Ethiopian national M&E program [33]. During recruitment, the age and sex of each of the students (recruitment data) were recorded. Students were asked to provide a fresh stool sample for 2 consecutive days. All stool samples were processed in the field using a duplicate Kato-Katz thick smear [16]. Fecal egg counts (FECs) expressed in eggs per gram of stool (EPG) were computed by multiplying the mean egg counts over 2 days by 24 for each of the STH species and *S*. *mansoni* separately.

Capillary blood was taken by finger prick with safety-lancets for capillary blood sampling (Safety-Lancet Extra 18G from Sarstedt). Approximately 0.5 to 1 mL capillary blood was collected from each student and plasma was separated in the field by centrifugation. Plasma samples from both the first (April 2019) and second (November 2019) survey were stored at -80°C at EPHI before shipment to the Laboratory of Parasitology at Ghent University (Belgium). All plasma samples were analyzed through the AsLungL3-ELISA assay based on the protocol described by Dana et al. (2020) using a 1:200 serum dilution and a 1:20,000 dilution of anti-human IgG4 as conjugate (SouthernBiotech) [30]. Reactivity to the AsLungL3 antigen was expressed in optical density ratio (ODr = (OD of sample–OD of negative control)/(OD of positive control–OD of negative control)). A collection of 91 blood samples from healthy Belgian blood donors, representing negative cases, were screened to determine the diagnostic cut-off. This cut-off was established as mean ODr + 3x standard deviation.

The first (April 2019) and second (November 2019) survey were conducted using the same approach except for data recording procedures. During the first part of the survey, all recruitment data (age and sex) and Kato-Katz thick smear results collected in the field were recorded on standardized data record forms and were thereafter registered by double data entry at EPHI. During the second part of the survey, recruitment data and Kato-Katz thick smear results were recorded through the SurveyCTO (Dobility, Inc.) mobile data collection platform, an android-based smartphone application that allows for standardized entry of data across all field teams.

### Statistical analysis

All analyses were performed in R studio (v1.3.1093) [37] and only include students for whom complete data were available for age, sex, and results on both diagnostic tests (Kato-Katz thick smear and AsLungL3-ELISA). Analyses were done on both school and woreda level. Ethiopia has three administrative levels; (1) regional states and chartered city administrations, (2) zones and (3) woredas (districts). These woredas are the implementation units for NTD control programs in Ethiopia. The woredas described in this study are in accordance with the national mapping of STHs in Ethiopia between 2013 and 2015, thus recent changes in administrative subdivisions were not considered [33].

The coproprevalence was calculated as the proportion of students for whom eggs in the stool were detected for any STH and the different STH species separately. The intensity of infection was determined based on the FEC thresholds recommended by the WHO, after which coproprevalence of moderate-to-heavy intensity (MHI) infections was calculated [17]. Mean FEC values were calculated per woreda and per school. The seroprevalence was defined as the proportion of students with an ODr on the AsLungL3-ELISA above the diagnostic cut-off. Mean ODr values were calculated per woreda and per school. All reported prevalence confidence intervals are 95% Wilson confidence intervals. The correlation between seroprevalence and mean ODr was investigated using the Spearman’s rank correlation test.

A generalized mixed logistic regression model was built by forward stepwise selection considering individual results. The AsLungL3-ELISA result (positive or negative) was used as a dependent variable while the parameters (1) age of student (in years), (2) sex of student (male or female), (3) *Ascaris* Kato-Katz thick smear result (positive or negative), (4) *Trichuris* Kato-Katz thick smear result (positive or negative) and all possible two-way interactions were tested as potential predicting variables. Hookworm was not included in this analysis due to the low reliability of hookworm egg counts. It has been shown that both (1) the time frame from production of stool to preparation of slides, and (2) the time frame from slide preparation to microscopic examination have a significant effect on the detection of hookworm eggs [38–40]. Although stool sample collection of 125 children in one school can be done within a reasonable short period of time, there are potential delays in refrigeration, processing of samples and microscopic examination of prepared Kato-Katz thick smears in the present survey due to the challenging fieldwork conditions. School ID (63 schools) and woreda ID (33 woredas) were implemented as random effects. For a simplified interpretation of the effect of age, the age was centralized around the median age (11 years).

Furthermore, the agreement between coproprevalence and seroprevalence in the classification of woredas for program decision-making was explored using the weighted Cohen’s Kappa Statistic (κ). This classification was based on the criteria proposed by the WHO during the implementation phase of a control program (prevalence ≥ 50%: 3x MDA/year; 50% > prevalence ≥ 20%: maintain MDA frequency; 20% > prevalence ≥ 10%: 1x MDA/year; 10% > prevalence ≥ 2%: 1x MDA/2 years; prevalence < 2%: no MDA) [15]. The value of this statistic indicates a slight (κ ≤ 0.2), fair (0.2 < κ ≤ 0.4), moderate (0.4 < κ ≤ 0.6), substantial (0.6 < κ ≤ 0.8) and an almost perfect agreement (κ > 0.8).

## Results and discussion

### Demographics of study population

Samples were collected from 6,718 students from 63 schools in 33 woredas across 7 regional states. Six of the initially selected schools (School IDs: 801, 802, 730, 727, 403, 408) and thereby 2 woredas were not sampled due to logistic and safety issues (**S1 Info**).

Subjects had a median age of 11 years old (25^th^ percentile: 10 years, 75^th^ percentile: 12 years). This median age varied across woredas from 10 to 16 years. Overall, the sexes were equally represented with a ratio of males to females of 1.12. This ratio varied per woreda from 0.51 to 4.15 with a median ratio of 1.02. The number of students screened per school ranged from 34 to 125, with a median of 117. **S2 Info** and **S3 Info** provide data on the number of subjects screened, the median age and the sex ratio across the 63 schools and 33 woredas.

### Coproprevalence

**Fig 1:**
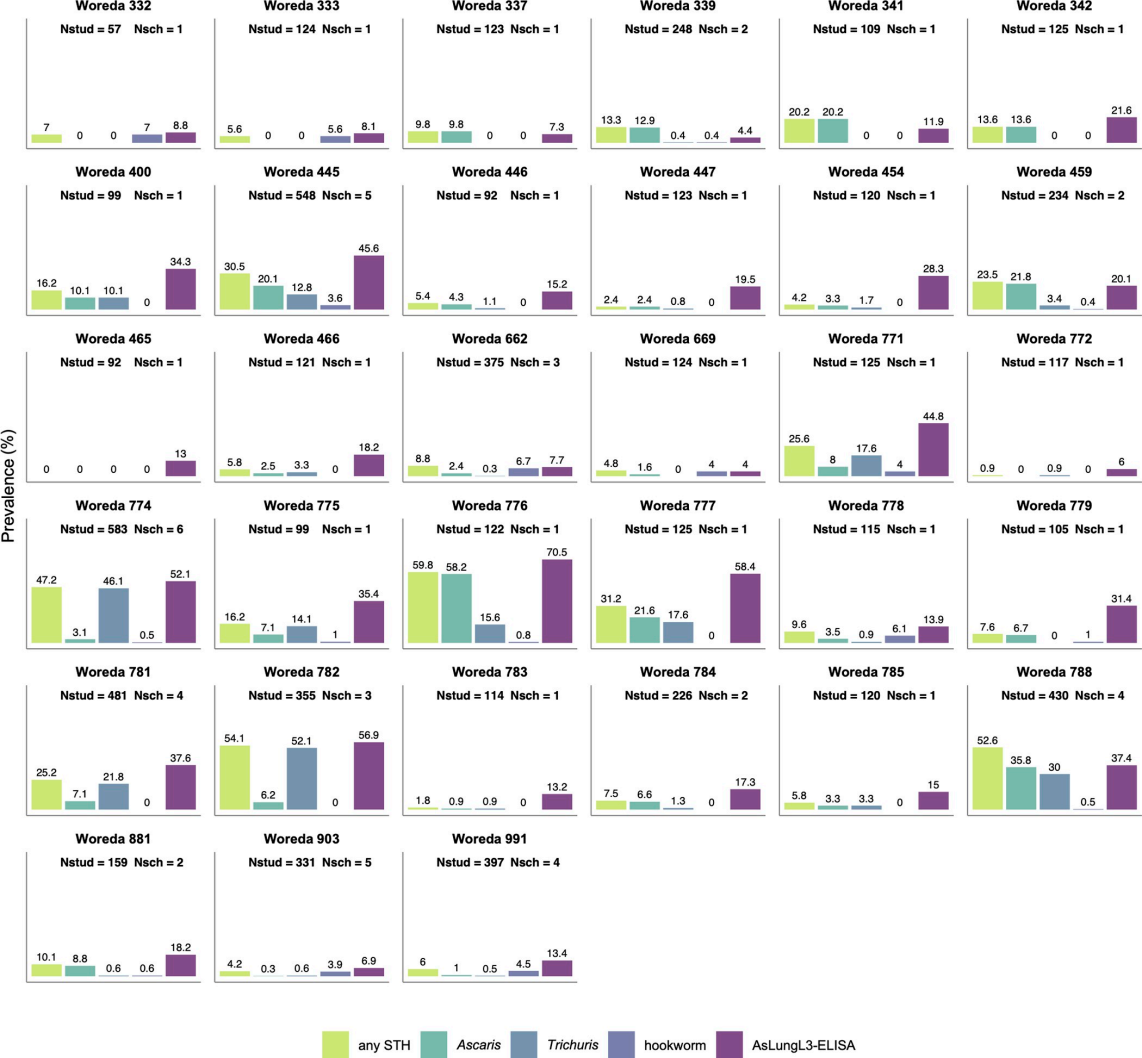
Coproprevalence and seroprevalence of STHs per woreda. The coproprevalence (any STH, *Ascaris*, *Trichuris* and hookworm) was calculated as the proportion of students for whom eggs in the stool were detected by Kato-Katz thick smear. The seroprevalence (AsLungL3-ELISA) was defined as the proportion of students with an optical density ratio (ODr) on the AsLungL3-ELISA above the diagnostic cutoff. Nstud: number of students per woreda, Nsch: number of schools per woreda. Samples were collected in woredas of the following 7 regional states: Amhara (woredas 332–342), Oromia (woredas 400–466), Benishangul-Gumuz (woredas 662 & 669), SNNPR (771–788), Gambella (woreda 881), Tigray (woreda 903) and Harari (woreda 991).

Overall, any STH eggs were observed in stool samples of 21.8% (95% CI 20.9–22.8) of the students, with *Trichuris* being the most prevalent STH species (prevalence 13.1%; 95% CI 12.3–13.9). The coproprevalence for *Ascaris* was 10.0% (95% CI 9.3–10.7), while this was 1.7% (95% CI 1.4–2.1) for hookworms. On a woreda level, the coproprevalence for any STHs ranged between 0.0 and 60.0% with a median of 9.6%. The coproprevalence per STH species, and for any STHs is summarized in **S2 Info** (school level) and **S3 Info** (woreda level). **Fig 1** gives a visual overview of coproprevalences in the 33 woredas.

### Choice of parameter to report serology results

**Fig 2:**
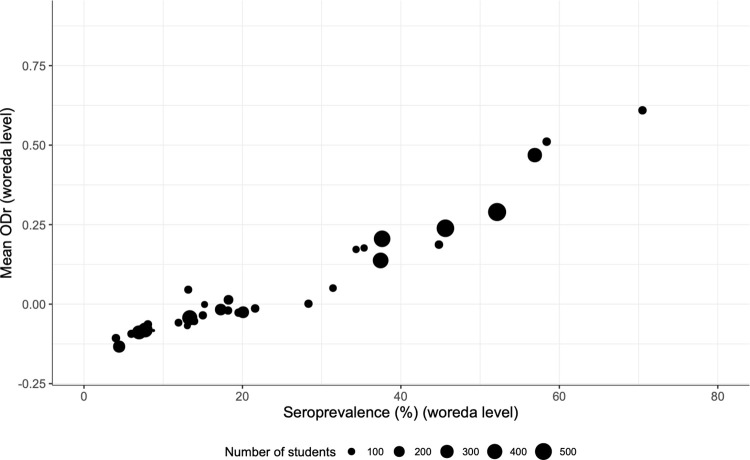
Correlation between seroprevalence and mean optical density ratio on woreda level. The size of the dots indicates the number of screened students per woreda. ODr: optical density ratio (ODr = (OD of sample–OD of negative control)/(OD of positive control–OD of negative control)).

After analysis of individual plasma samples on the AsLungL3-ELISA, the serology results on woreda level can be presented as either the percentage seropositive individuals (seroprevalence) or as mean ODr value. With the objective to define the preferred serological parameter for further analyses, both variables were calculated for the 33 sampled woredas. The two parameters were highly correlated as indicated in **Fig 2** (ρ 0.96, p-value < 2.2e-16). In a field study in 101 Belgian pig farms, Vlaminck and colleagues observed a similar trend in the relationship between seroprevalence and mean ODr measured by the AsHb-ELISA [24]. Considering this correlation, seroprevalence was chosen as parameter to report serology results in this study, in agreement with the current use of coproprevalence as parameter for coprology. It has to be stated that there is no evidence that seroprevalence outperforms mean ODr as a parameter to report serology results. Yet, the use of seroprevalence facilitates a comparison between serology and coprology based on the percentages currently used for program decision-making (2%, 10%, 20% and 50%). Accordingly, individual results were handled qualitatively (positive or negative) in further analyses.

The linear correlation between seroprevalence and mean ODr is different to what is observed in coprology data where the relationship between the parameter coproprevalence and the parameter mean FEC of infected individuals (parameter used for worm burden) is far from linear [41]. **S4 Info** shows the relationship between coproprevalence and mean FEC of infected individuals observed in the present survey for both *Ascaris* and *Trichuris*. It was seen that on woreda level, similar mean FECs go along with a broad range in coproprevalence. Furthermore, it is known that large changes in mean FEC are sometimes only reflected by very small changes in coproprevalence [41]. Therefore, the use of coproprevalence data for the M&E of an STH control program might be questioned since this data does not immediately reflect a drop or rise in environmental contamination with worm eggs. Environmental contamination is a key aspect in the epidemiology of STH species as transmission occurs through their infectious stages in the soil, and the level of exposure to the parasites will impact the morbidity due to migrating immature stages. This is in sharp contrast to serological data, reflecting an immune response of the host triggered by exposure to the infectious stages. Indeed, an experimental infection study with *Ascaris* in pigs provided evidence that the AsLungL3-ELISA was able to measure a seroconversion for which the extent of the antibody response (ODr value) was fully dependent on the infection dose given [25]. On top of that, since there is evidence from the veterinary field that increased anti-*Ascaris* antibody levels are correlated with increased liver pathology and reduced growth rates, the potential to link serology to assessments of child growth and cognitive development (e.g. height, weight, BMI, cognitive skills) might be a turning point for research into the real disease burden caused by soil-transmitted helminthiasis [24, 27, 28].

### Seroprevalence

**Fig 1** gives a visual overview of seroprevalence together with coproprevalence of any STH and the different STH species separately in the 33 woredas. Seroprevalence on woreda level ranged from 4.0 to 70.5% with a median of 18.2%. All serology data is summarized in **S2 Info** (school level) and **S3 Info** (woreda level). In general, the proportion of children in this study that developed an antibody response was higher than the proportion of children excreting worm eggs. Diverse biological and epidemiological aspects are at the base of this observation, which is in line with previous studies in both animals and humans [24, 30, 31]. First, due to innate and acquired immune responses of the host, only a small percentage of all immature worms eventually develops into mature adult worms. Indeed, for *Ascaris*, a self-cure expulsion reaction taking place 14 to 21 days post-infection eliminates most larvae from the small intestine of the host [42]. Secondly, it can take several weeks or months, depending on the species, before worms are sexually mature and start shedding eggs. During this prepatent period, false negative coprology results will be recorded. Thirdly, egg production by adult worms varies largely due to density dependent factors, male/female worm ratios, geography and the immune status of the host [19–23]. Hence, the number of eggs in the stool is not linearly correlated with the number of adult worms in the intestines, neither is the number of adult worms in the intestines representative for the level of exposure to infectious stages.

### Cross-reactivity of the AsLungL3-ELISA

The outcome of the logistic regression model, considering individual results, indicated a significant effect of the variables (1) sex (p-value < 0.001), (2) *Ascaris* Kato-Katz thick smear result (p-value = 0.002) and (3) *Trichuris* Kato-Katz thick smear result (p-value = 0.039) on the AsLungL3-ELISA outcome (positive or negative). There were no significant two-way interaction terms. At the reference age of 11 years (median age), the odds for testing positive on the AsLungL3-ELISA among students having a positive *Ascaris* Kato-Katz thick smear result was 1.37 times (95% CI 1.12–1.67) the odds among students testing negative for *Ascaris* on Kato-Katz thick smear (37% higher). In the case of *Trichuris*, the odds for a positive AsLungL3-ELISA test result for students testing positive on Kato-Katz thick smear was 1.20 (95% CI 1.01–1.43) times the odds for students testing negative on Kato-Katz thick smear (20% higher). These results suggest cross-reactivity of individuals harbouring patent *Trichuris* infections to the AsLungL3 antigens. In a previous cross-sectional study of Dana et al. (2020), measuring anti-AsLungL3 antibody responses in 1,200 children and 600 adults in an endemic region in Ethiopia, the authors wanted to evaluate cross-reactivity by categorizing individuals according to the type of patent STH infection, but antibody responses were very similar across the different groups. Therefore, it was not possible to draw conclusions with regards to the species-specificity of the assay [30]. Very recently, the results of a longitudinal study in 66 Ethiopian children of 4–6 years of age suggested that there was no or only a limited level of cross-reactivity to AsLungL3 antigens due to *Trichuris* infections [32]. However, in that longitudinal study, the cohort of children that remained both *Ascaris* negative over the course of the survey and were positive for *Trichuris* or hookworm at least once was very small (*Trichuris* n = 26, hookworm n = 5) [32]. In pigs, cross-reactivity of the AsHb-ELISA has already been observed. Samples from pigs infected with very high numbers of *Trichuris suis* eggs (3,000) showed an increased reactivity [24]. While the AsHb-ELISA is based on a single native antigen (*A*. *suum* haemoglobin) purified from the pseudocoelomic fluid of adult *Ascaris* worms, the AsLungL3-ELISA is based on an antigen mixture of which AsHb is expected to be a component [43]. The fact that the AsLungL3-ELISA makes use of a complete larval extract instead of a specific purified antigen increases the likelihood that antibodies to antigens of other pathogens can cross-react.

**Fig 3:**
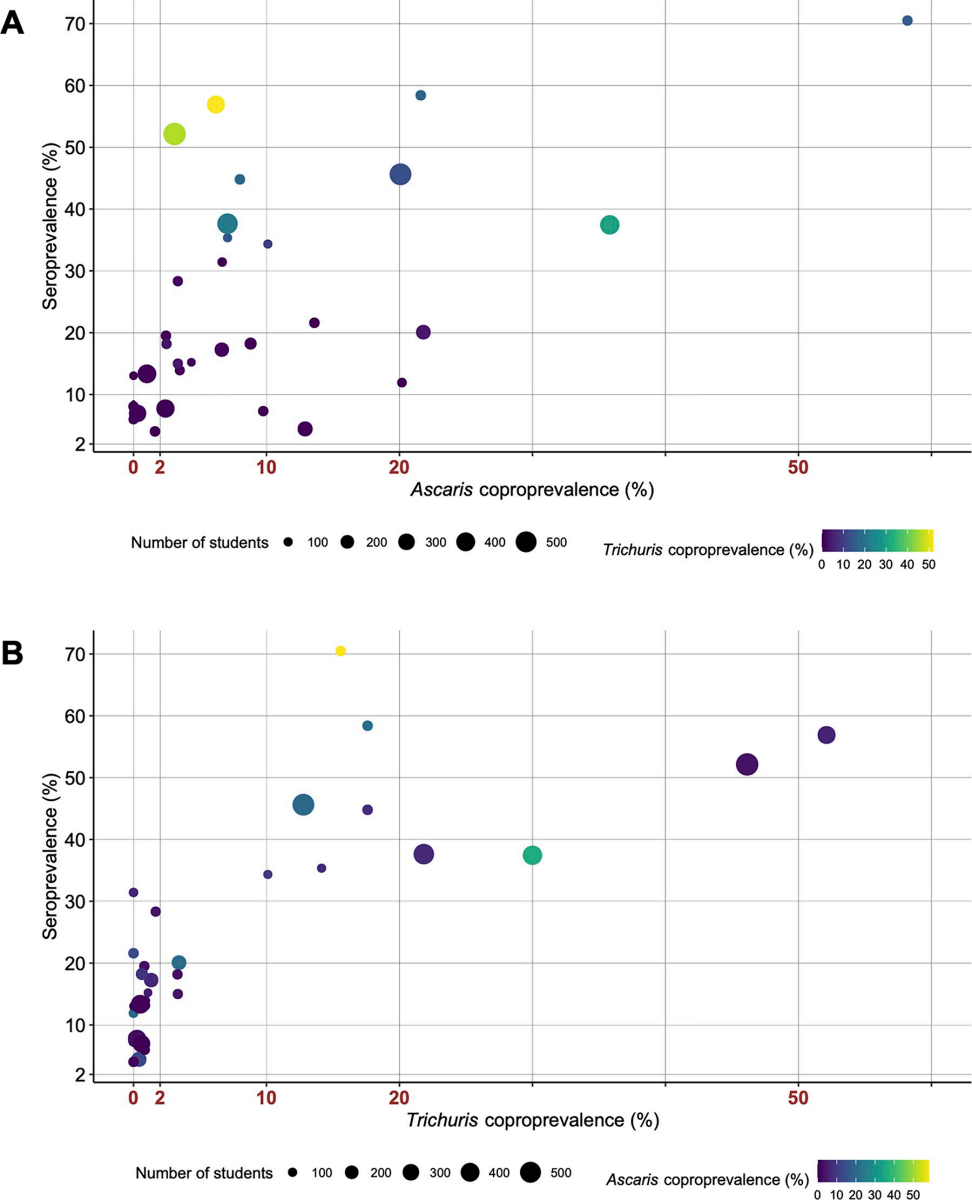
Correlation between coproprevalence and seroprevalence on woreda level. **Panel A** shows the correlation between *Ascaris* coproprevalence and seroprevalence, including *Trichuris* prevalence of the woredas as indicated by their color. **Panel B** displays the correlation between *Trichuris* coproprevalence and seroprevalence, showing *Ascaris* coproprevalence by color scale. The WHO coproprevalence thresholds for program decision-making regarding the frequency of drug administration are indicated in red on the x-axis [15]. The size of the dots indicates the number of screened students per woreda.

This cross-reactivity is also apparent in **Fig 3**. **Fig 3A** shows *Ascaris* coproprevalence on the x-axis while *Trichuris* coproprevalence is indicated by a color scale. For most woredas, seroprevalence tends to be exceeding coproprevalence. Remarkably, two woredas in SNNPR (774 and 782) showed a considerable higher seroprevalence than *Ascaris* coproprevalence. Their colors (light green and yellow) indicate a very high prevalence of patent *Trichuris* infections, thus confirming the result of the logistic regression model. Indeed, **Fig 3B** shows that woredas surpassing a *Trichuris* coproprevalence of 10% have a considerable proportion of individuals testing positive on the AsLungL3-ELISA, regardless of *Ascaris* endemicity. However, a vast number of woredas with *Trichuris* coproprevalence below 2% record a much higher seroprevalence, reflecting the presence of *Ascaris*.

In pig production systems, limited cross-reactivity is no restricting factor to the use of the AsHb and AsLungL3-ELISA assays [24,25]. This is because *A*. *suum* is by far the most prevalent parasite in modern pig farms [44]. On the contrary, in the scope of developing new diagnostic assays for human STHs, the TPPs define a test highly specific for each parasite species [12]. This prerequisite is based on substantial evidence acquired through simulation studies showing that specificity is the most important diagnostic parameter when the prevalence drops [45–47]. The low specificity of the AsLungL3-ELISA might be a concern when aiming for prevalence estimates for the different STH species separately. On the contrary, a pan-helminth biomarker detecting multiple STH species might be of particular interest for making stop program decisions or in the framework of non-stool based diagnostic platforms to be implemented in post-program surveillance. A possible way forward is therefore the identification of immunogenic antigens in the larval extract based on plasma of either *Ascaris*- or *Trichuris*-infected individuals, which would allow for species-specific test development. Moreover, this can provide an answer to the question whether epitopes present in the extract that evoke specific antibody production are of protein or glycan nature. In the present study, screened students might additionally have been exposed to other endemic helminth species for which diagnosis was not performed such as *Strongyloides stercoralis*, *Enterobius vermicularis*, *Onchocerca volvulus* and *Wuchereria* spp. or *Brugia* spp. Although, comparison of the AsLungL3 seroprevalence with onchocerciasis and lymphatic filariasis endemicity in Ethiopia did not indicate any cross-reactivity of the AsLungL3-ELISA due to the latter three [35,36]. There was no cross-reactivity due to *S*. *mansoni*, the only non-STH species investigated (**S6 Info**). Besides, a study of Dana et al. (2020) showed no cross-reactivity due to exposure to *Toxocara* spp. [30]. The call for species-specific and/or pan-helminth biomarkers stresses the need for well-characterized biobanks containing serum samples that are much better defined [48]. To date, FIND takes up a leading role in biobank services, with a network of integrated biobanks storing more than 400,000 samples to support the development of tools improving the diagnosis of infectious diseases in low- and middle-income countries (https://www.finddx.org). Furthermore, FIND hosts a virtual biobank providing a global view of infectious disease collections managed by organizations worldwide (https://vbd.finddx.org). Currently, there is no FIND biobank that includes characterization of samples regarding STH infections, and this might be an important step in streamlining the development of new STH diagnostics. As recently discussed by Vlaminck and colleagues, blood samples could be collected from different populations: longitudinal samples in endemic areas from subjects with changing infection status (strategy 1), samples from young children in areas endemic for only a single STH (strategy 2), samples from experimentally infected non-endemic individuals (strategy 3) or samples from experimentally infected animals using the original or a closely related parasite species (strategy 4) [48]. Sampling strategy 1 was recently applied by Dana and colleagues, indeed resulting in important insights into the potential of serodiagnostics in young children [32]. Sampling strategy 2 might be utmost challenging since tropical regions are often endemic for multiple STH species, next to other NTDs, not only from parasitic origin. There are historic and recent studies performing experimental infections in humans (strategy 3) but upscaling such trials for the development of diagnostics would face many challenges [49–52]. On the contrary, animal models can play a pivotal role to assess the performance of new diagnostic tests since animals experimentally infected with a single STH species represent the ultimate gold standard [24, 25, 53–57]. In addition, these models allow an evaluation of the potential of new tools to diagnose different phases of the helminth’s life cycle, this might be of key interest for STH species considering the fact that morbidity is caused by both adult worms residing in the intestines and immature stages migrating through the body of the host.

### Comparison between coproprevalence and seroprevalence to guide decision-making in STH control programs

**Fig 4:**
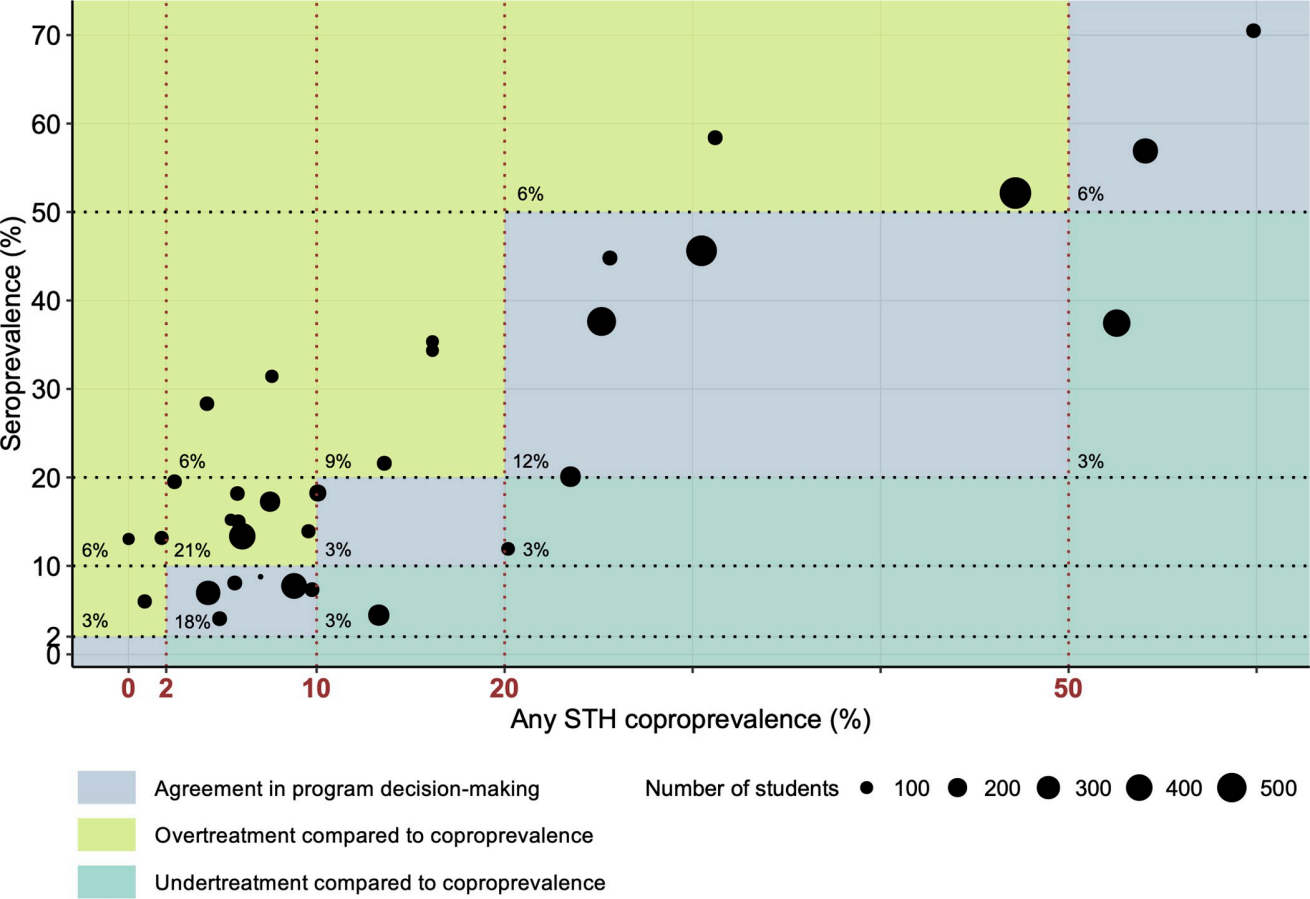
Program decision-making based on any STH coproprevalence and seroprevalence. Classification based on the program decision tree endorsed by the WHO during the implementation phase of a control program (prevalence ≥ 50%: 3x MDA/year; 50% > prevalence ≥ 20%: maintain MDA frequency; 20% > prevalence ≥ 10%: 1x MDA/year; 10% > prevalence ≥ 2%: 1x MDA/2 years; prevalence < 2%: no MDA) [15]. The WHO coproprevalence thresholds are indicated in red on the x-axis. The same thresholds were extrapolated to the seroprevalence data due to a lack of evidence-based alternatives. The size of the dots indicates the number of screened students per woreda. The percentage of woredas ranked in a certain category is shown in the left bottom corner.

To date, periodic follow-up surveys measuring any STH coproprevalence are used to adapt the frequency of drug administration to present epidemiological situations. The decision tree endorsed by the WHO contains any STH coproprevalence decision-making thresholds to be used at the start of the program (prevalence ≥ 50%: 2x MDA/year; 50% > prevalence ≥ 20%: 1x MDA/year; prevalence < 20%: no MDA) and during the implementation phase (prevalence ≥ 50%: 3x MDA/year; 50% > prevalence ≥ 20%: maintain MDA frequency; 20% > prevalence ≥ 10%: 1x MDA/year; 10% > prevalence ≥ 2%: 1x MDA/2 years; prevalence < 2%: no MDA) [15]. Currently, there are no defined thresholds for STH prevalence measured by serology. Such seroprevalence thresholds are already operative in program decision-making for onchocerciasis; less than 0.1% seropositivity measured by IgG4 ELISA to the *O*. *volvulus* Ov16 antigen in children aged <10 years is currently recommended for stopping MDA [58]. As well as for lymphatic filariasis with WHO guidelines for discontinuing MDA recommending a threshold of <2% antigenemia (<1% if *Aedes* is the main vector) assessed by the filariasis test strip detecting *Wuchereria bancrofti* circulating filarial antigen and <2% seropositivity on antibody assays detecting IgG4 to *Brugia* spp. [59]. To address the main research question of this study namely ‘Is there agreement in program decision-making based on seroprevalence and coproprevalence?’, classification of the woredas was done following the WHO implementation phase program decision-making thresholds for both any STH coproprevalence and seroprevalence. In other words, the coproprevalence thresholds (2%, 10%, 20% and 50%) were extrapolated to the seroprevalence data due to a lack of well-defined alternatives. Thus, there is no evidence that these thresholds are the most appropriate values to perform program decision-making based on serology. Likewise, it must be pointed out that there is no evidence that seroprevalence outperforms mean ODr as a parameter to guide decision-making, yet the use of prevalence results facilitated a comparison between the two tests. The weighted kappa statistic between the tests was calculated to be 0.43 indicating moderate agreement. As illustrated in **Fig 4**, for about 39% of the woredas (13/33) there was agreement in program decision-making (blue), 51% (17/33) of the woredas was ranked in a category that would lead to a higher treatment frequency in comparison to coproprevalence (green), while only 9% (3/33) of the woredas would be treated less frequently (turquoise). Very importantly, only 4 of the 33 woredas were assigned to a non-adjacent category, and this only for coproprevalences beneath 10%. To complete the comparison between serology and coprology to guide decision-making, **S5 Info** illustrates their relationship based on the parameter mean ODr instead of the parameter seroprevalence.

In summary, using the same threshold values (2%, 10%, 20%, 50%) for seroprevalence predominantly leads to the implementation of a higher frequency of drug administration. This observation can be explained by biological and epidemiological factors discussed earlier with serology being a measurement of exposure and coprology a parameter for patent infection. In addition, it is possible that there is a difference in the effect of MDA on the reduction in seroprevalence compared to the reduction in coproprevalence [30,31]. Unfortunately, the present dataset does not allow to include MDA frequency in the analyses since no serological examination was done during the national mapping between 2013 and 2015 (baseline). An analogous longitudinal study would allow to study the effect of MDA frequency on seroprevalence, mean ODr and coproprevalence and to perform subgroup analysis for the agreement in program decision-making. Next to these factors, the typical occurrence of discrepancy in classification for low prevalence regions can also be a result of the smaller intervals (e.g., interval 2 to 10% compared to interval 20 to 50%). Here one could question whether the performance (reproducibility, sensitivity and specificity) of an egg-based test is high enough to overcome this issue. In addition, since in most surveys students are asked to provide a stool sample themselves, there is no absolute certainty about the origin of the screened samples. This contrasts with the collection of finger prick blood where standardized subject recruitment forms and accurate labelling of samples assure up to 100% traceability, provided that field teams received adequate training. Not only performance of the diagnostic test and reliability of sample origin, but also survey design (number of clusters and subjects per cluster) and spatial heterogeneity (intra-cluster correlation e.g. within a school) have a significant impact on program decision-making [47]. Aiming to further refine serology-specific thresholds, either based on seroprevalence or on mean ODr, to be used in program decision-making requires a larger dataset than the one currently available. Analogous to the present study for which schools with a different history in parasite prevalence were selected, the dataset needs to include regions with a broad range in coproprevalence for all different STH species. A first possible strategy to compile this dataset is applying serology in nationwide M&E efforts of national STH control programs in countries different from Ethiopia, the study site of this case study. A second and undoubtedly most interesting possible pathway is the implementation of serology in large-scale intervention trials like the Geshiyaro Project (Ethiopia; launched in 2019) [60] and DeWorm3 (Benin, India and Malawi; started in 2015) [61,62]. Both studies test the feasibility of interrupting the transmission of STHs by applying one or multiple interventions such as (1) MDA, (2) water, sanitation and hygiene (WaSH) and (3) behaviour change communication (BCC). It is stated that transmission interruption is achieved if less than 2% community prevalence is measured by qPCR. This value is based on transmission models suggesting that reaching a 2% prevalence of any STH 24 months after stopping MDA reliably predicts transmission interruption with a positive predictive value of >90% [63, 64]. Next to monitoring the trends in seroprevalence and mean ODr in such trial settings, a main added value of combining forces between intervention trials and serology studies could be the establishment of a serology-specific threshold defining the transmission breakpoint in agreement with 2% prevalence measured by qPCR, thereby creating unique opportunities to validate the capacity of serology for making stop treatment and surveillance decisions.

## Conclusion

This is the first time that serology for soil-transmitted helminthiasis is applied on such extensive scale; a nationwide survey embedded in a control program context with the collection of data from more than 6,700 students. Three research questions were addressed. Firstly, qualitative (seroprevalence) and quantitative (mean ODr) serology results were highly correlated, and considering the current use of coproprevalence for program decision-making, seroprevalence was selected as parameter to report serology results. Secondly, the results of the present study suggested cross-reactivity of the AsLungL3-ELISA due to *Trichuris* infections. Further research should therefore focus on the optimization of either a species-specific or pan-helminth serodiagnostic assay, both of particular interest regarding different potential applications of serology in program decision-making (e.g. stop intervention decisions, post-program surveillance). Thirdly, using the same WHO threshold values (2%, 10%, 20% and 50%) for coproprevalence and seroprevalence, decisions based on the latter predominantly led to the implementation of a higher frequency of drug administration. Thus, further refinement of serology-specific program decision-making thresholds is needed. To conclude, the present study is a proof-of-concept that serology holds promise as alternative tool to monitor and evaluate STH control programs.

## Supporting information

S1 Info Maps of Ethiopian sentinel schools selected for this study. **Panel A**: 14 geographical clusters of sentinel schools with varying levels of *Ascaris* endemicity were purposively selected considering logistic and safety concerns, resulting in a total of 69 schools across 35 woredas in 7 regional states. **Panel B**: 63 of these schools (in 33 woredas across 7 regional states) were successfully sampled (red). Six of the initially selected schools (School IDs: 801, 802, 730, 727, 403, 408) and thereby 2 woredas were not sampled due to logistic and safety issues (green). Maps were made using QGIS (version 3.16.16) [34]. The base layer image was obtained from OpenStreetMap (http://www.openstreetmap.org) which is made available under the Open Data Commons Open Database License (https://www.openstreetmap.org/copyright). The source of the administrative boundaries was www.gadm.org/.
(PDF)

S2 Info School level data. All selected schools are listed. Geographical information includes coordinates (latitude, longitude and altitude (if available)), regional state and woreda. Survey information consists of survey date, cluster number (**S1 Info**) and field team ID. *Ascaris* endemicity level for the purpose of the school selection (level 1: coproprevalence = 0%; level 2: 0% < coproprevalence < 5%; level 3: 5% ≤ coproprevalence < 10%; level 4: 10% ≤ coproprevalence < 20%; level 5: 20% ≤ copro-prevalence < 30%; level 6: copro-prevalence ≥ 30%) was calculated based on coproprevalence results for *Ascaris* during the national mapping of STHs in Ethiopia between 2013 and 2015 [33]. Endemicity of lymphatic filariasis and onchocerciasis is documented on woreda level based on data of Sime et al. (2018) and Meribo et al. (2017) [35, 36]. Results of the 2019 survey include the detected coproprevalence for any STH and the different STH species for both any intensity and moderate-to-heavy intensity (MHI) infections as well as the coproprevalence for *Schistosoma mansoni*. Seroprevalence based on the AsLungL3-ELISA is recorded. All prevalence confidence intervals given are 95% Wilson score intervals. Mean FECs and mean ODr values on school level are documented. Furthermore, the number of students screened, the median age and the ratio male/female per school are recorded.
(XLSX)

S3 Info Woreda level data. Endemicity of lymphatic filariasis and onchocerciasis is documented based on data of Sime et al. (2018) and Meribo et al. (2017) [35, 36]. [35, 36]. Results of the 2019 survey include the detected coproprevalence for any STH and the different STH species for both any intensity and moderate-to-heavy intensity (MHI) infections as well as the coproprevalence for *Schistosoma mansoni*. Seroprevalence based on the AsLungL3-ELISA is recorded. All prevalence confidence intervals given are 95% Wilson score intervals. Mean FECs and mean ODr values on woreda level are documented. Furthermore, the number of students screened, the median age and the ratio male/female per woreda are recorded.
(XLSX)

S4 Info Relationship between mean fecal egg count of infected individuals and coproprevalence observed in the present survey. **Panel A**: *Ascaris*. **Panel B**: *Trichuris*. The size of the dots indicates the number of screened students per woreda.
(PDF)

S5 Info Correlation between any STH coproprevalence and mean ODr on woreda level. The WHO coproprevalence thresholds for program decision-making regarding the frequency of drug administration are indicated in red on the x-axis [15]. The size of the dots indicates the number of screened students per woreda.
(PDF)

S6 Info Correlation between *Schistosoma mansoni* coproprevalence and seroprevalence on woreda level. Any STH coproprevalence of the woredas is included by a color scale. The WHO coproprevalence thresholds for program decision-making regarding the frequency of drug administration are indicated in red on the x-axis [15]. The size of the dots indicates the number of screened students per woreda.
(PDF)
