# Supplementary material for: Comparison of coproprevalence and seroprevalence to guide decision-making in national soil-transmitted helminthiasis control programs: Ethiopia as a case study
Source: PLoS Negl Trop Dis. 2022 Oct 5;16(10):e0010824. doi: 10.1371/journal.pntd.0010824 (PMC9534397; doi:10.1371/journal.pntd.0010824)

S1 Info Maps of Ethiopian sentinel schools selected for this study

Maps were made using QGIS (version 3.16.16) (https://qgis.org/en/site/). The base layer image was obtained from OpenStreetMap (http://www.openstreetmap.org) which is made available under the Open Data Commons Open Database License (https://www.openstreetmap.org/copyright). The source of the administrative boundaries was www.gadm.org/.

A

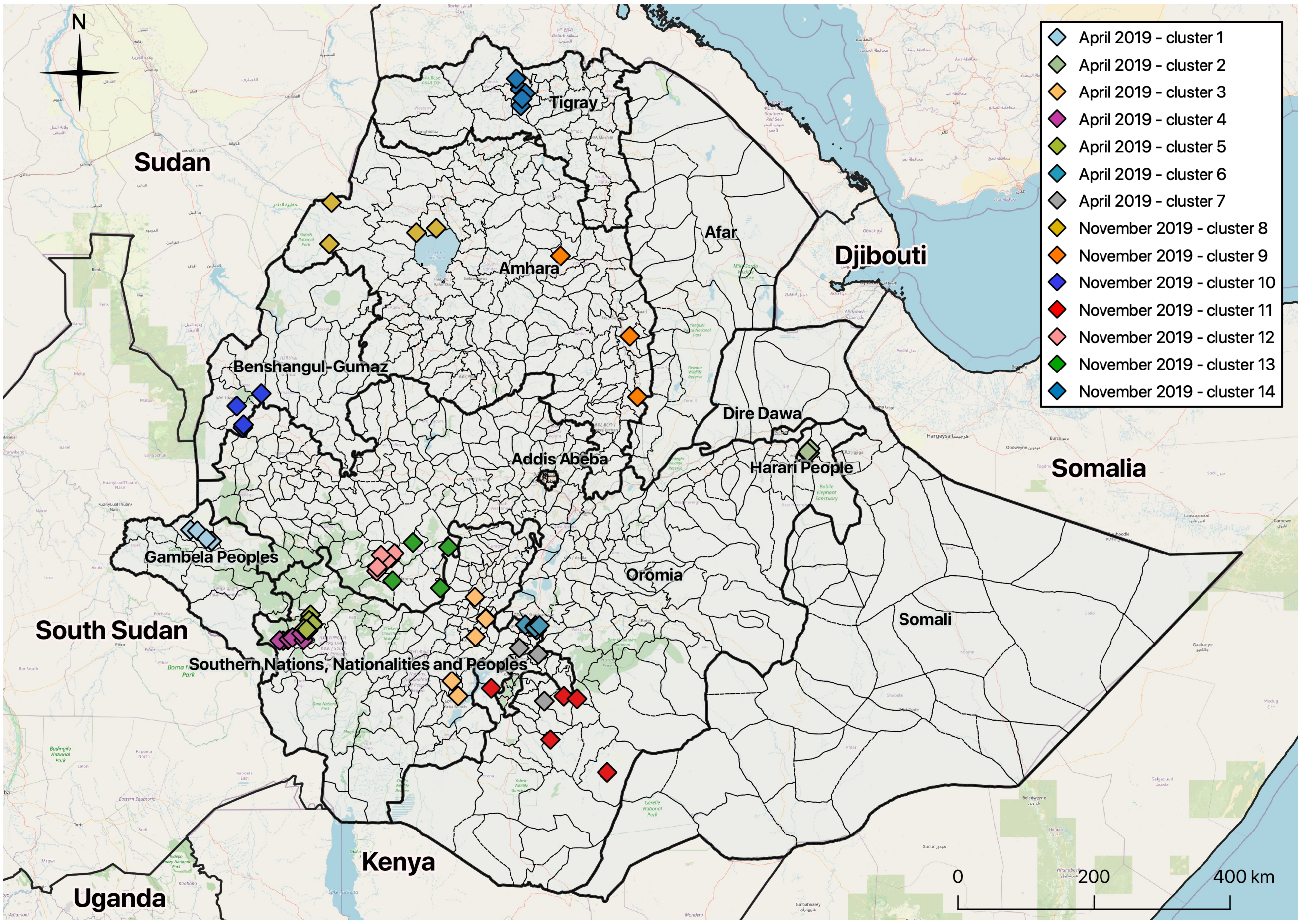

B

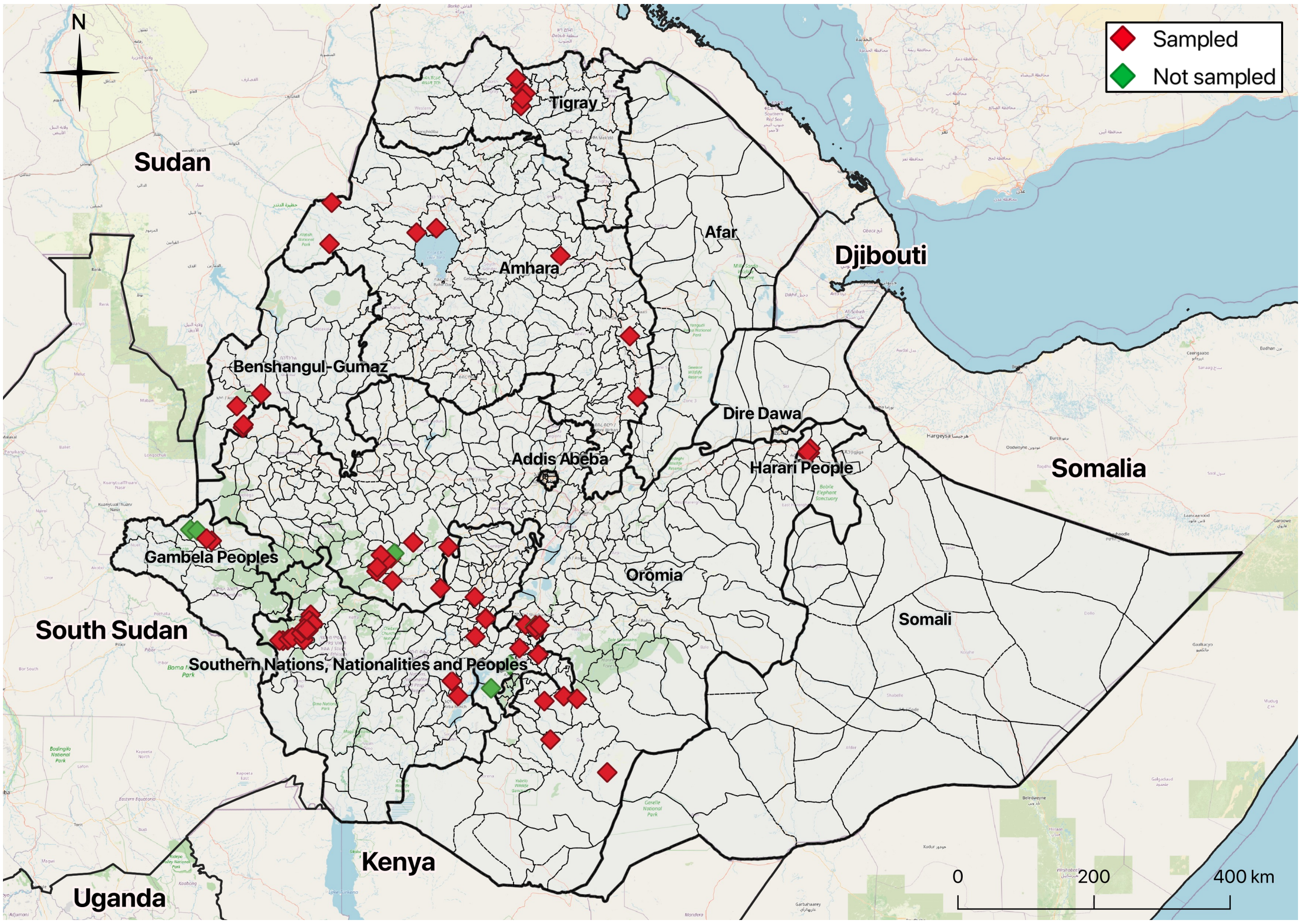

Supplement: S1 Info — Panel A: 14 geographical clusters of sentinel schools with varying levels of Ascaris endemicity were purposively selected considering logistic and safety concerns, resulting in a total of 69 schools across 35 woredas in 7 regional states. Panel B: 63 of these schools (in 33 woredas across 7 regional states) were successfully sampled (red). Six of the initially selected schools (School IDs: 801, 802, 730, 727, 403, 408) and thereby 2 woredas were not sampled due to logistic and safety issues (green). Maps were made using QGIS (version 3.16.16) [34]. The base layer image was obtained from OpenStreetMap (http://www.openstreetmap.org) which is made available under the Open Data Commons Open Database License (https://www.openstreetmap.org/copyright). The source of the administrative boundaries was www.gadm.org/. (PDF) [file pntd.0010824.s001.pdf]
