# Supplementary material for: Comparison of coproprevalence and seroprevalence to guide decision-making in national soil-transmitted helminthiasis control programs: Ethiopia as a case study
Source: PLoS Negl Trop Dis. 2022 Oct 5;16(10):e0010824. doi: 10.1371/journal.pntd.0010824 (PMC9534397; doi:10.1371/journal.pntd.0010824)

<u>S4 Info</u>

Relationship between mean fecal egg count of infected individuals and coproprevalence observed in the present survey for both *Ascaris* (panel A) and *Trichuris* (panel B)

A

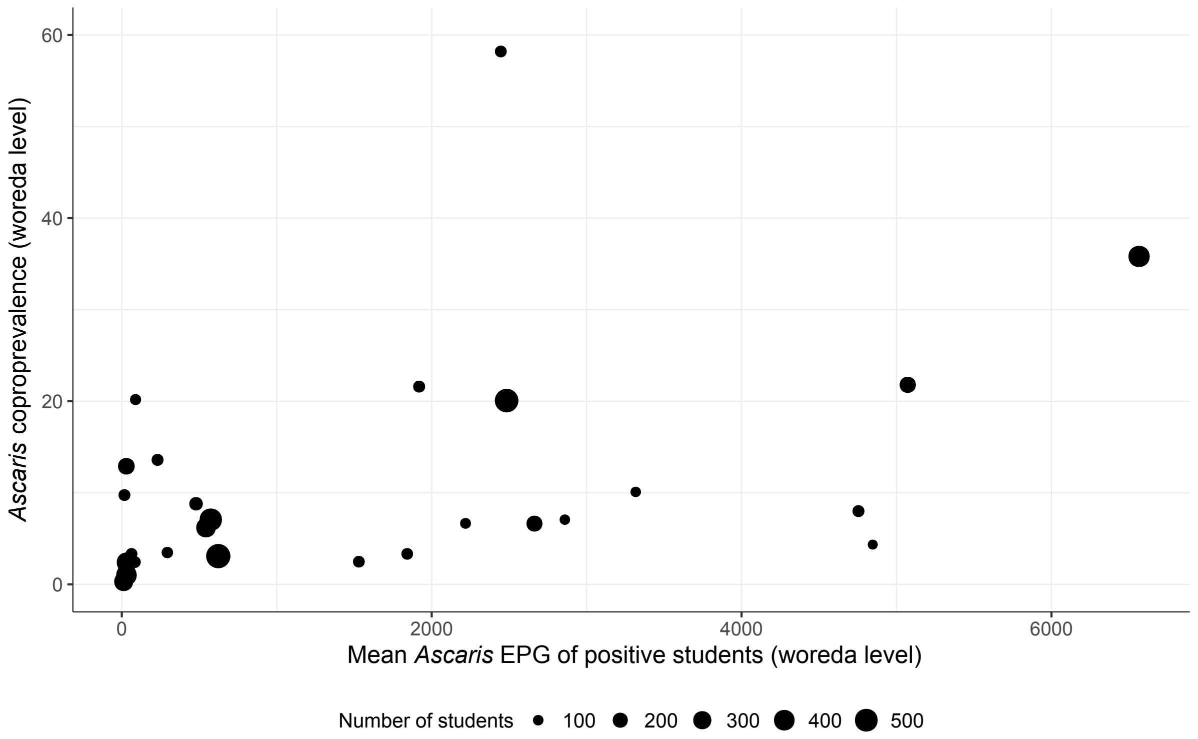

B

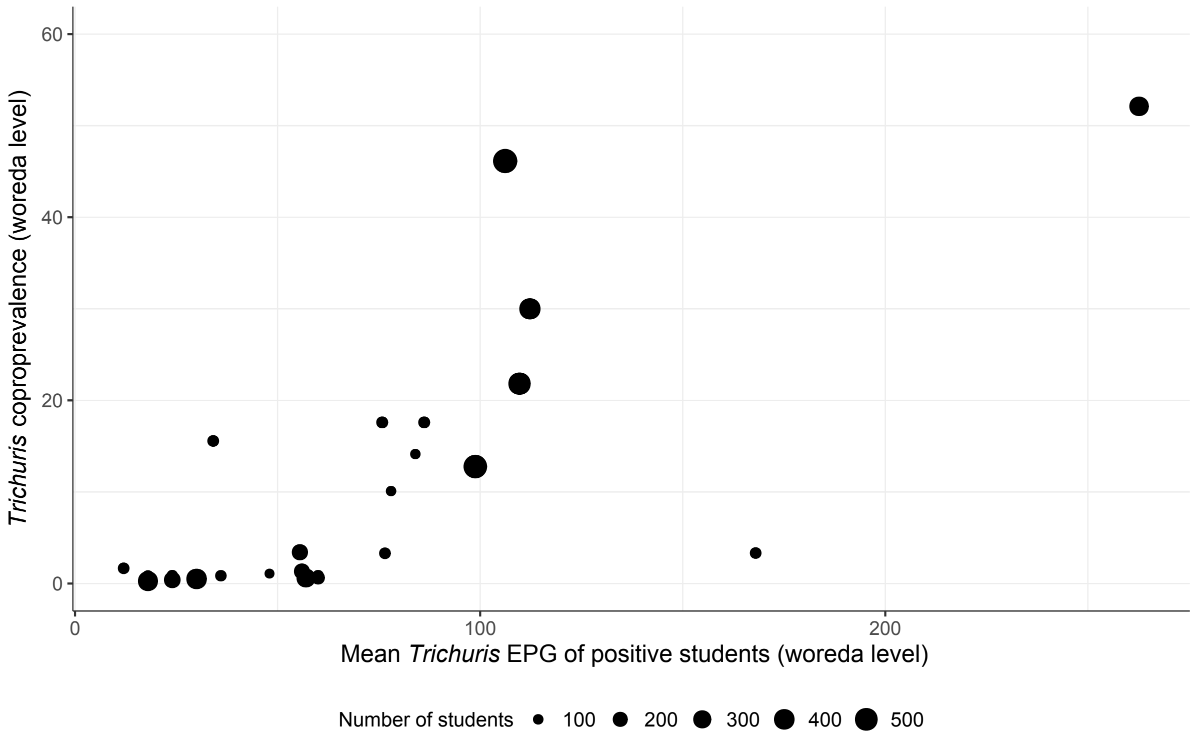

Supplement: S4 Info — Panel A: Ascaris. Panel B: Trichuris. The size of the dots indicates the number of screened students per woreda. (PDF) [file pntd.0010824.s004.pdf]
