# Supplementary figures and images for: Comparison of coproprevalence and seroprevalence to guide decision-making in national soil-transmitted helminthiasis control programs: Ethiopia as a case study
Source: PLoS Negl Trop Dis. 2022 Oct 5;16(10):e0010824. doi: 10.1371/journal.pntd.0010824 (PMC9534397; doi:10.1371/journal.pntd.0010824)

### S5 Info

Correlation between any STH coprovalence and mean ODr on woreda level

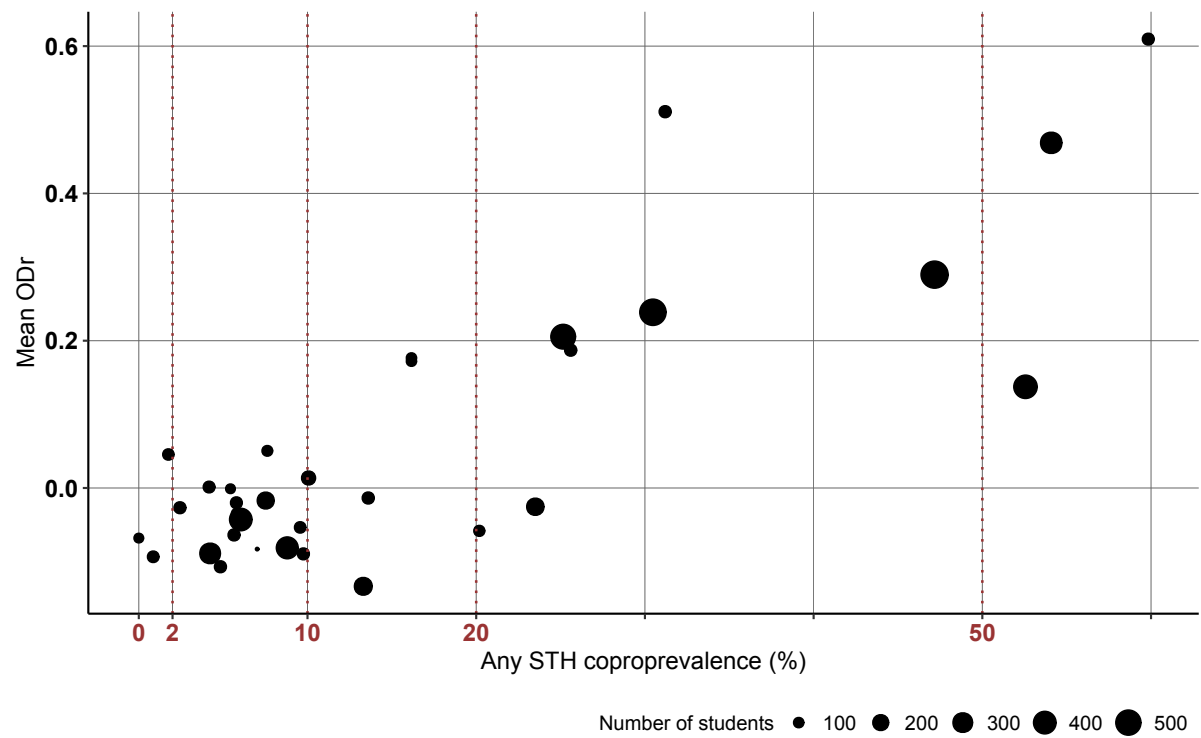

Supplement: S5 Info — The WHO coproprevalence thresholds for program decision-making regarding the frequency of drug administration are indicated in red on the x-axis [15]. The size of the dots indicates the number of screened students per woreda. (PDF) [file pntd.0010824.s005.pdf]
